# Letter to “Cultural adaptation and reliability assessment of the Hammersmith neonatal neurological examination for Brazilian newborns at risk of cerebral palsy”

**DOI:** 10.1055/s-0045-1802309

**Published:** 2025-02-06

**Authors:** Tathiana Ghisi de Souza

**Affiliations:** 1 Faculdade de Medicina de Jundiaí, Departamento de Pediatria, Jundiaí SP, Brazil.; 2 Universidade Estadual de Campinas, Faculdade de Ciências Médicas, Departamento de Pediatria, Campinas SP, Brazil.; 3 Protocolos de Avaliação Neonatal e Infantil de Hammersmith (HNNE-HINE), Campinas SP, Brazil.

Dear Editors,

It was with great interest and appreciation that I read the article by Correr and Pfeirfer, 1 which reported the translation of the Hammersmith Neonatal Neurological Examination (HNNE). I would like to express some considerations and my concerns regarding the article published on pages 47 to 54, volume 81, of the journal *Arquivos de Neuro-Psiquiatria* . While reading it, I have identified some significant errors in its methodology and content.

I have noted that the part that addresses the scales contains incorrect information. For example, the neonatal evaluation (HNNE) should be performed on babies from 0 to 1-month-old, and on premature babies from 40 weeks of gestational age up to the first month, while the HINE evaluation is indicated for babies from 2 to 24 months. 2 3 In addition, the dates of publication of the scales are not correct. The abridged version cited in the article is from a 2005 study by Mercuri et al., 4 and is not intended for screening purposes, like the 2012 check list published by Romeo et al. 5 This mistake can lead to confusion and inappropriate use of the form.

I would also like to point out that Fig. 1 1 does not correspond to the cited scale, but rather to the upper half of the complete HNNE front page. Furthermore, the assessment can be translated, but it does not require cultural adaptation, validation, or psychometric analysis, since it is not a psychometric test but rather a neurological assessment. It is unclear why Brazilian babies would behave differently from babies from other countries. What do they mean by the “cross-adaptation process”? And “adapting it to the Brazilian culture”?

It is important to note that interexaminer agreement was partly assessed through filming. However, the HNNE should not be interpreted through filming, since it is essential for the evaluator to feel the baby's response during the assessment. Furthermore, the methodology of psychometric analysis to obtain validity evidence is outdated and, sometimes, incorrect. 6 One example is the statistical test used to determine interexaminer agreement. Since questionnaires or scales are ordinal variables and not continuous ones, the use of the intraclass correlation coefficient (ICC) is not appropriate.

The translation was not made available as supplementary material in the article, and the inconsistency in the explanations for application makes me question whether the exam was performed correctly, also considering the errors in the methodology and data analysis.

These errors concern me, since the assessment in question is receiving some attention, and such errors can result in confusion and inappropriate use of the assessment, leading to incorrect prognoses.

I acknowledge that the liability for the published articles' content lies with the authors themselves. However, in writing this letter, my goal is not to question the responsibility of the Journal or its editorial board, but to draw the attention of the scientific community to these issues.

As an instructor and the first Hammersmith assessments translator in Brazil, I understand the complexity in finding clear explanations about scales that do not require practical certification for professional use. However, it is essential to guarantee the evaluators' experience, which makes all the difference in the results interpretation, especially in the neonatal period. The publication errors do not clarify this experience on the part of the authors and evaluators.
